# CD36 upregulates DEK transcription and promotes cell migration and invasion via GSK-3β/β-catenin-mediated epithelial-to-mesenchymal transition in gastric cancer

**DOI:** 10.18632/aging.103985

**Published:** 2020-11-21

**Authors:** Jin Wang, Ti Wen, Zhi Li, Xiaofang Che, Libao Gong, Zihan Jiao, Xiujuan Qu, Yunpeng Liu

**Affiliations:** 1 Department of Medical Oncology, The First Hospital of China Medical University, Shenyang 110001, China; 2 Key Laboratory of Anticancer Drugs and Biotherapy of Liaoning Province, The First Hospital of China Medical University, Shenyang 110001, China; 3 Liaoning Province Clinical Research Center for Cancer, The First Hospital of China Medical University, Shenyang 110001, China; 4 Key Laboratory of Precision Diagnosis and Treatment of Gastrointestinal Tumors, Ministry of Education, The First Hospital of China Medical University, Shenyang 110001, China

**Keywords:** CD36, epithelial-to-mesenchymal transition, gastric cancer, DEK

## Abstract

Evidence indicates that the lipid scavenger receptor CD36 has pro-metastatic functions in several cancers. Although CD36 expression correlates with an unfavorable prognosis in gastric cancer (GC), its specific contribution to disease onset, progression, and/or metastasis remains unclear. Using bioinformatics analyses, we ascertained that CD36 expression was increased in metastatic GC specimens in The Cancer Genome Atlas and Gene Expression Omnibus databases and correlated with poor prognosis. In addition, higher CD36 expression was associated with lymph node metastasis (*p* < 0.05) and poor prognosis (*p* = 0.030) in 79 Chinese GC patients. Basal CD36 expression levels correlated positively with migration, invasion, and expression of epithelial-to-mesenchymal transition (EMT) markers in GC cell lines, a relationship confirmed by knockdown and overexpression experiments. Importantly, analysis of gene expression changes in CD36-knockdown GC cells led us to identify the chromatin-associated protein DEK as a c-Myc target that mediates activation of the GSK-3β/β-catenin signaling pathway to trigger EMT. These findings further our understanding of the mechanisms governing metastatic dissemination of GC cells and suggest the therapeutic potential of strategies targeting CD36.

## INTRODUCTION

Gastric cancer (GC) is a common malignancy worldwide. Two-thirds of GC patients are diagnosed with advanced-stage disease and present metastatic dissemination, which contributes to a high mortality rate [1–3]. Although research has provided compelling insights into the progression of GC, the mechanisms responsible for metastatic spreading remain unclear. The metastatic process occurs through a multi-step cascade which is closely related to the molecular alterations present in both cancer cells and the tumor microenvironment [4–6]. Among these alterations, reprogramming of metabolic pathways has been highlighted in recent years as a critical process leading to the malignant progression of cancer cells [7, 8]. Particularly, molecular abnormalities related to lipid metabolism have shown to provide suitable conditions for proliferation, invasion, and angiogenesis in many tumor types [9].

CD36 is a transmembrane protein involved in the transport and metabolism of certain lipids, such as long-chain fatty acids and oxidized low-density lipoproteins, and can also exert different signal transduction functions through its intracellular domains [10, 11]. Studies have highlighted the pro-tumorigenic and pro-metastatic actions of CD36 in various cancer types, including oral squamous cell carcinoma, breast cancer, and melanoma [12]. However, due to its multi-functional role, the potential metastasis-promoting activity of CD36 in GC and other cancers remains to be fully characterized [13].

In multiple cancers, including GC, epithelial-to-mesenchymal transition (EMT) is a crucial multi-step process through which tumor cells acquire metastatic potential [14]. Although CD36 expression has been correlated with EMT and metastasis in different tumor types (*e.g.* oral squamous cell carcinoma, hepatocellular cancer, and cervical cancer), the specific mechanisms remain controversial [11, 12, 15, 16]. Elevated CD36 expression predicts poor prognosis in GC [17]. However, to the best of our knowledge no studies have explored the role of CD36 on EMT in GC cells. Therefore, we conducted gene expression analysis in both public GC databases and an internal GC patient cohort, and carried out *in vitro* experiments to evaluate the impact of CD36 overexpression on GC cell migration and invasion, as well as its potential role in EMT.

## RESULTS

### High CD36 expression is associated with poor clinicopathological outcome in GC patients

**Figure 1:**
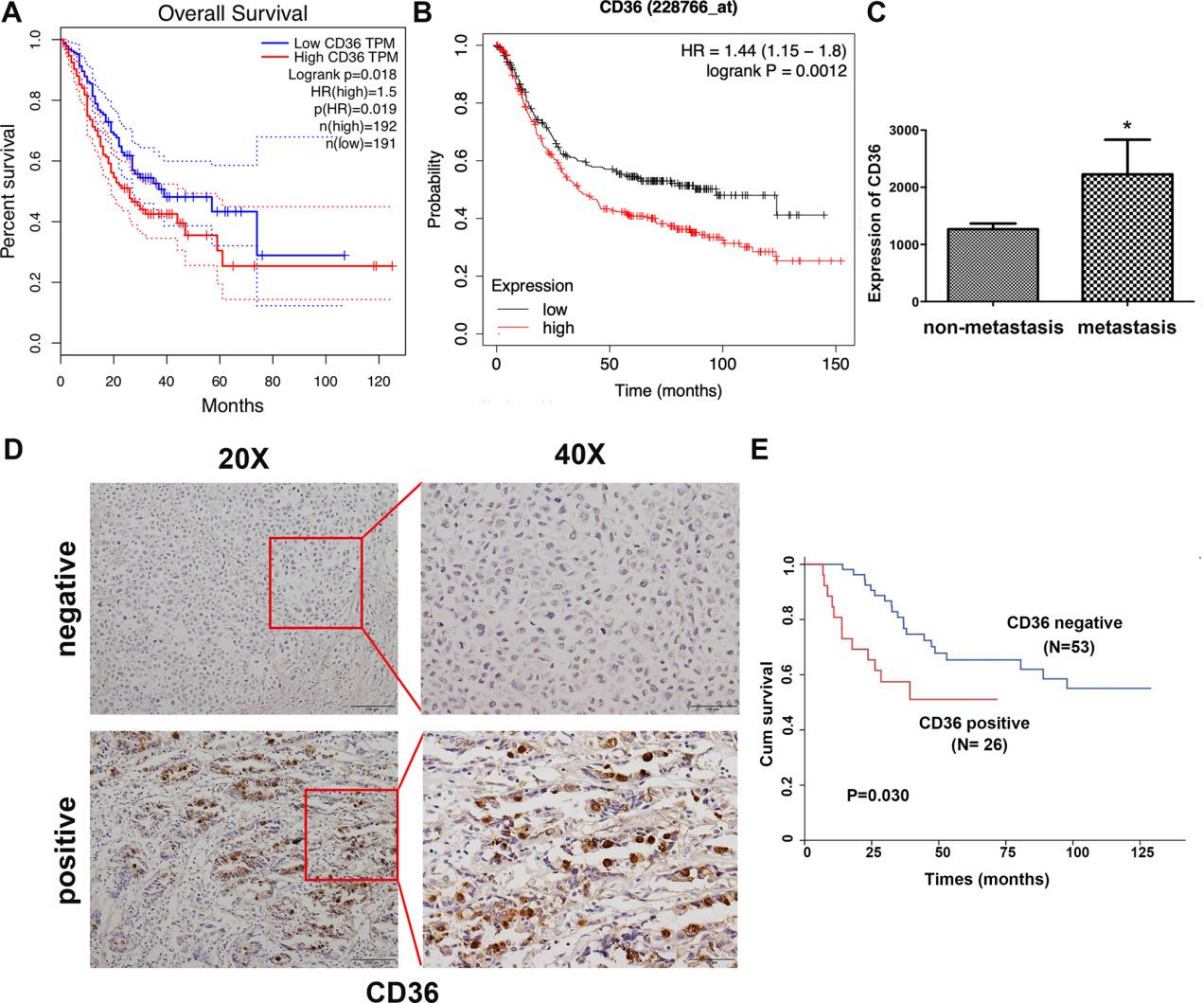
**Increased CD36 expression is associated with poor clinicopathological features and clinical outcomes in GC patients.** (**A**) Kaplan-Meier analysis of OS based on CD36 expression in GC patients in TCGA database. (**B**) Kaplan-Meier analysis of OS based on CD36 expression in GC patients in the GEO database. (**C**) Analysis of CD36 expression in non-metastatic and metastatic GC samples in TCGA database. *p < 0.05 vs. non-metastasis group. (**D**) Representative images of CD36 IHC in GC specimens. Top: negative CD36 expression; bottom: positive CD36 expression; left: IHC images, 200×; right: IHC images, 400×. (**E**) Association between CD36 expression and overall survival in 79 GC patients (*p* = 0.030).

Correlation analysis conducted on clinical GC samples from TCGA and GEO databases showed that high expression of CD36 is associated with poor patient prognosis (Figure 1A, 1B). In turn, compared to non-metastatic specimens, higher CD36 expression was detected for metastatic GC in TCGA database (Figure 1C). Subsequently, we detected the expression of CD36 in 79 GC patients via immunohistochemistry (IHC). Results showed that CD36 localized both in the cytoplasm and on cellular membranes (Figure 1D). Negative expression was found in 53 cases (67.1%), whereas positive expression was detected in 26 cases (32.9%). An evaluation of the correlation between CD36 expression and various clinicopathological parameters revealed a higher CD36 expression rate in lymph node-positive patients (Table 1). Furthermore, GC patients with high CD36 expression had worse survival outcomes (*p* = 0.030, Figure 1E).

**Table 1:** Correlations between CD36 expression and clinicopathological features in 79 GC patients.

**Variable**	**Cases**	**CD36 expression (%)**		**P value**
		**Negative**	**Positive**	
Gender				0.543
Male	51	33(62.3%)	18(69.2%)	
Female	28	20(37.7%)	8(30.8%)	
Age				0.795
<60	47	31(58.5%)	16(61.5%)	
≥60	32	22(41.5%)	10(38.5%)	
T stage				0.696
T1/T2/T3	31	20(37.7%)	11(42.3%)	
T4a/T4b	48	33(62.3%)	15(57.7%)	
Lymph node metastasis				0.041
Absence	34	27(50.9%)	7(26.9%)	
Presence	45	26(49.1%)	19(73.1%)	
Differentiation				0.950
Poor	49	33(62.3%)	16(61.5%)	
Moderate	30	20(37.7%)	10(38.5%)	

### CD36 promotes migration and invasion by regulating EMT in GC cells

**Figure 2:**
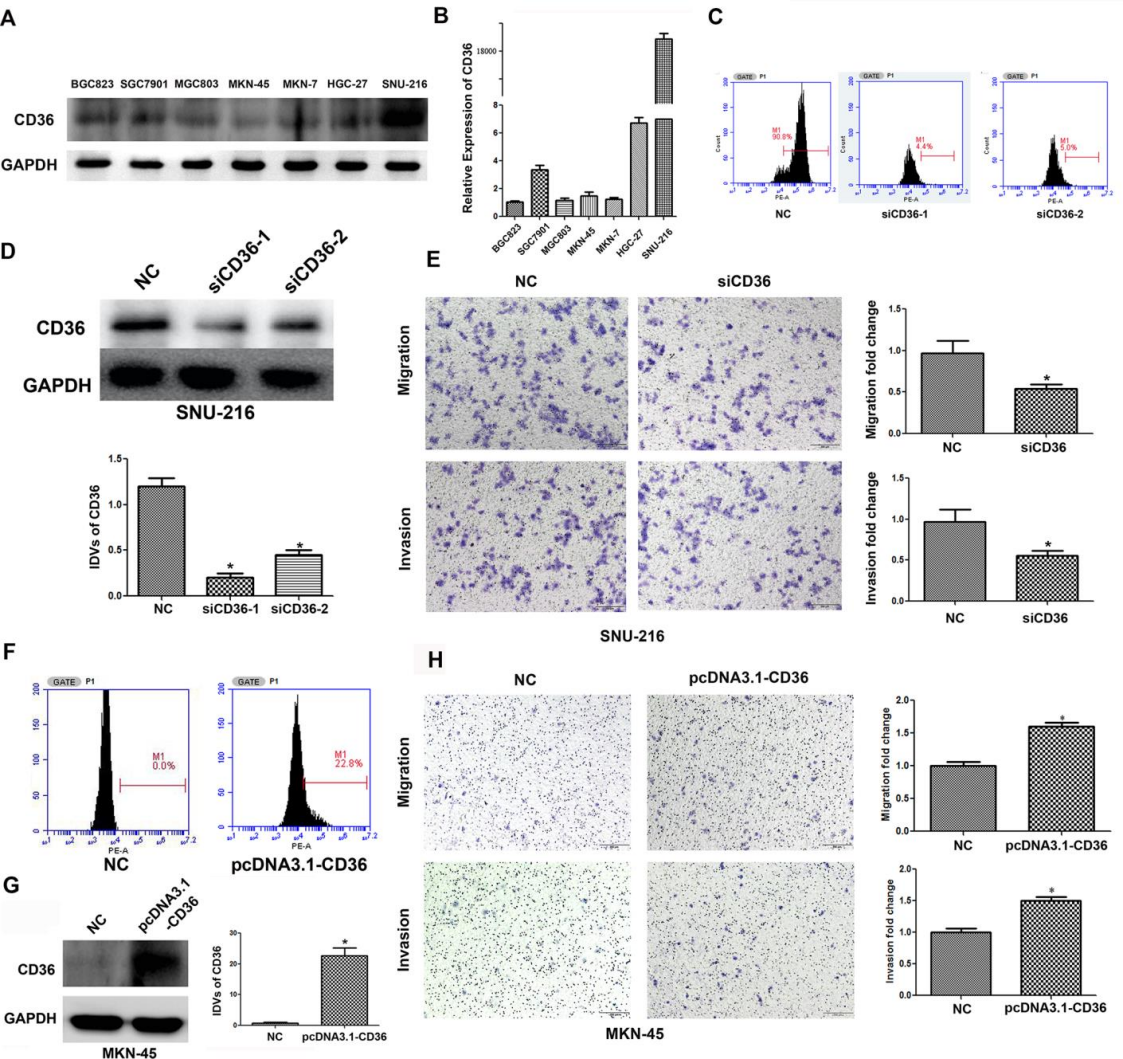
**CD36 promotes migration and invasion in GC cells.** (**A**) Analysis of CD36 protein expression in GC cell lines. (**B**) Analysis of CD36 mRNA expression in GC cell lines. (**C**) Flow cytometric analysis of the transfection efficiencies of NC/siCD36 in SNU-216 cells. (**D**) Western blot analysis of the transfection efficiencies of NC/siCD36 in SNU-216 cells. (**E**) Results of Transwell migration and invasion assays in SNU-216 cells transfected with NC/siCD36. (**F**) Flow cytometric analysis of the transfection efficiencies of NC/pcDNA3.1-CD36 in MKN-45 cells. (**G**) Western blot analysis of the transfection efficiencies of NC/pcDNA3.1-CD36 in MKN-45 cells. (**H**) Results of Transwell migration and invasion assays in MKN-45 cells transfected with NC/pcDNA3.1-CD36. Data are presented as the mean ± SD. *p < 0.05 vs. NC group. GAPDH was used as endogenous control in western blot assays. IDV: integrated densitometric value.

**Figure 3:**
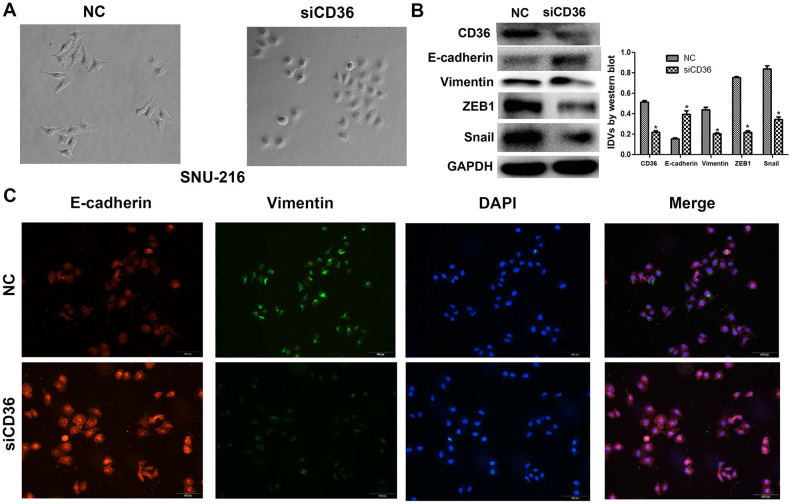
**CD36 knockdown inhibits EMT in GC cells.** (**A**) Morphological changes in SNU-216 cells transfected with NC/siCD36 (phase contrast microscopy, 200×). (**B**) Western blot analysis of EMT markers in SNU-216 cells transfected with NC/siCD36. Data are presented as the mean ± SD. *GAPDH was used as endogenous control.* (**C**) Immunofluorescence staining for E-cadherin and vimentin in SNU-216 cells transfected with NC/siCD36. *p < 0.05 vs. NC group.

To investigate the effects of CD36 expression in GC, flow cytometry, qRT-PCR, and western blot analyses were first applied to characterize the expression of CD36 in seven GC cell lines (BGC823, SGC7901, MGC803, MKN-45, MKN-7, HGC-27, and SNU-216). Results showed that CD36 was expressed at the highest level in SNU-216 cells, while the lowest expression was detected in MKN-45 cells (Figure 2A, 2B and Supplementary Figure 1A). Therefore, SNU-216 and MKN-45 cells were selected to assess their migration and invasion potential by Transwell assays. The results showed that SNU-216 cells had stronger migration and invasive abilities than MKN-45 cells (Supplementary Figure 1B). To evaluate whether CD36 expression would impact these results, we performed siRNA-mediated CD36 silencing in SNU-216 cells (Figure 2C, 2D) and plasmid-mediated CD36 overexpression (pcDNA3.1-CD36) in MKN-45 cells (Figure 2F, 2G). Compared to the negative siRNA control (NC) group, siCD36-transfected SNU-216 cells showed significantly decreased migration and invasion abilities (Figure 2E). Conversely, MKN-45 cells transfected with pcDNA3.1-CD36 showed enhanced migration and invasion (Figure 2H). These data indicate that CD36 expression promotes cell migration and invasion in GC cells. Notably, we observed that following CD36-knockdown, SNU-216 cells modified their characteristic spindle-like fibroblastic morphology and adopted the cobblestone-like appearance typical of epithelial cells (Figure 3A). This change suggests that CD36 silencing reverses EMT in GC cells [18].

Having shown a close association between CD36 expression and GC cell migration and invasion, we further evaluated whether the expression of EMT markers (E-cadherin and vimentin) and EMT-related transcription factors (finger E-box binding homeobox 1, ZEB1, and Snail) was affected by CD36 depletion in SNU-216 cells. Western blot results indicated that the expression of E-cadherin was markedly increased, while the expression of vimentin, ZEB1, and Snail was decreased in siCD36-transfected cells (Figure 3B). These findings were further supported by immunofluorescence assays, which showed, in comparison to the NC siRNA group, increased expression of E-cadherin and decreased expression of vimentin in SNU-216 cells transfected with siCD36 (Figure 3C). These data further suggest that CD36 promotes migration and invasion in GC cells by inducing EMT.

### CD36 positively regulates DEK to promote migration and invasion in GC cells

**Figure 4:**
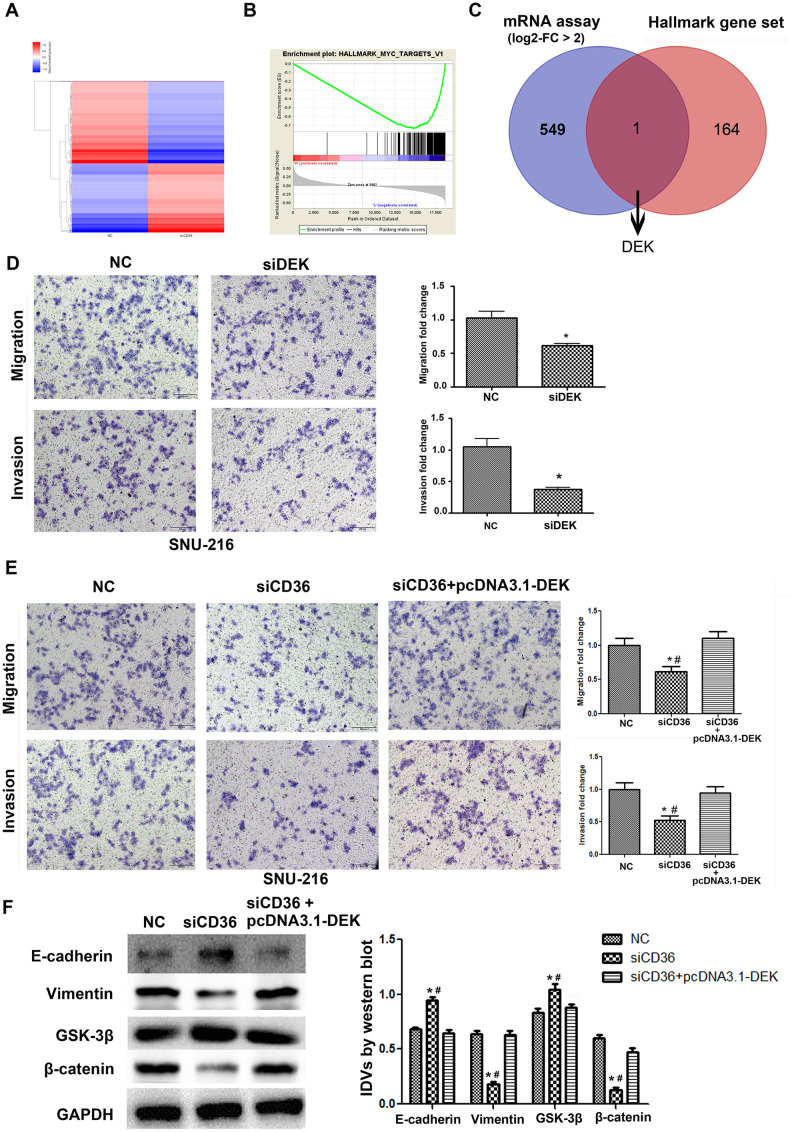
**CD36 positively regulates DEK to promote migration and invasion in GC cells.** (**A**) Heatmap of log2-fold gene expression changes in SNU-216 cells transfected with NC/siCD36. (**B**) Enrichment of genes within the Myc-targets-V1 pathway in the CD36-down-regulated Hallmark gene set. (**C**) Venn diagram of candidate genes downstream of CD36 obtained from combined mRNA array and MSigDB Hallmark-GSEA analyses. (**D**) Results of Transwell migration and invasion assays conducted on SNU-216 cells transfected with NC/siDEK. (**E**) Results of Transwell migration and invasion assays carried out in SNU-216 cells transfected with NC/siCD36/siCD36 + pcDNA3.1-DEK. (**F**) Western blot analysis of EMT and GSK-3β/β-catenin signaling markers in SNU-216 cells transfected with NC/siCD36/siCD36 + pcDNA3.1-DEK. Data are presented as the mean ± SD. For D-F, *p < 0.05 vs. NC group; #*p* < 0.05 vs. siCD36 + pcDNA3.1-DEK group. GAPDH was used as endogenous control.

To further validate the promoting effect of CD36 on GC cell migration and invasion, a microarray was used to screen for differential mRNA expression in SNU-216 cells transfected with siCD36. Compared to cells transfected with NC siRNA, 550 down-regulated genes (log2-fold change > 2, data not shown) were identified in siCD36-transfected cells (Figure 4A). Through GSEA analysis, the most significant MSigDB Hallmark gene set pathway identified in correlation with CD36 downregulation (i.e. Myc-Targets-V1 pathway) was used to further screen potential regulators of CD36 functions in GC cells (Figure 4B). One mRNA transcript, corresponding to the DEK proto-oncogene, was thus selected for further analysis (Figure 4C). Transwell assays showed that si-RNA-mediated DEK knockdown impaired migration and invasion potential in SNU-216 cells (Figure 4D). However, this effect was reversed following co-transfection with siCD36 and pcDNA3.1-DEK (Figure 4E). Furthermore, this maneuver rescued also the expression of both EMT and GSK-3β/β-catenin signaling markers (Figure 4F). These results suggested that CD36 promotes migration and invasion of GC cells by up-regulating DEK expression.

### CD36 mediates c-Myc-induced DEK transcription in GC cells

**Figure 5:**
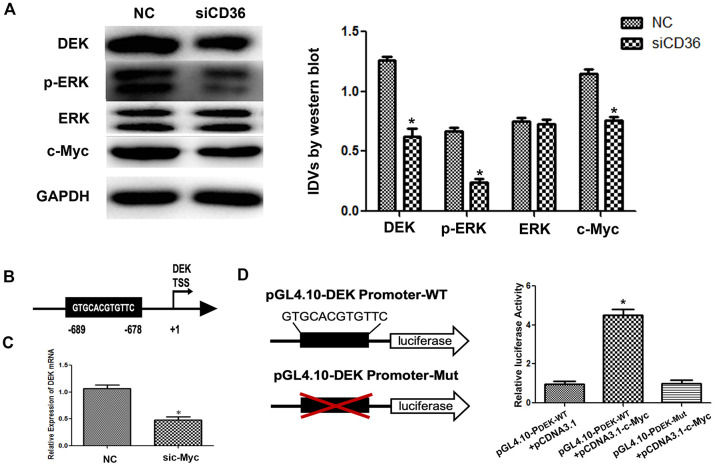
**CD36 promotes c-Myc-dependent DEK transcription in GC cells.** (**A**) Western blot analysis in SNU-216 cells transfected with NC/siCD36. *p < 0.05 vs. NC group. GAPDH was used as endogenous control. (**B**) Schematic diagram of the DEK gene promoter; the c-Myc-binding site is highlighted. (**C**) Analysis of DEK mRNA expression following siRNA-mediated c-Myc knockdown (sic-Myc) in SNU-216 cells. *p < 0.05 vs. NC group. (**D**) Dual luciferase assay results from SNU-216 cells co-transfected with firefly luciferase constructs (pGL4.10-DEK Promoter-WT or pGL4.10-DEK Promoter-Mut) and pcDNA3.1-c-Myc. Left: firefly luciferase constructs; right: relative luciferase activity. *p < 0.05 vs. GL4.10-DEK Promoter-WT + pcDNA3.1 group. *All data are presented as the mean ± SD.*

Previous studies have shown that CD36 promotes nuclear translocation of the transcription factor c-Myc after activation of ERK1/2 [19]. Western blot analysis showed that the expression of p-ERK1/2 in SNU-216 cells was significantly decreased following CD36 knockdown (Figure 5A). In turn, analysis of transcription factor binding profiles in the JASPAR database revealed the presence of a c-Myc-binding site (GTGCACGTGTTC) in the DEK promoter region [20] (Figure 5B). To verify that c-Myc activity regulates DEK expression, we examined whether changes in the corresponding protein levels occur in SNU-216 cells transfected with siCD36. Significantly decreased expression was detected for both c-Myc and DEK in western blot analysis (Figure 5A). In turn, qRT-PCR assays showed that siRNA-mediated c-Myc silencing resulted in decreased DEK transcription (Figure 5C). Subsequently, we generated DEK promoter-driven luciferase reporter vectors containing wild-type (WT) or mutant (Mut) c-Myc-binding sites. The luciferase reporter assay results indicated that c-Myc activated luciferase transcription in the presence of WT, but not Mut, DEK promoters (Figure 5D). These results indicate that CD36 upregulates DEK transcription in GC cells through c-Myc activation.

### CD36-mediated DEK upregulation enhances GSK-3β/β-catenin signaling in GC cells

**Figure 6:**
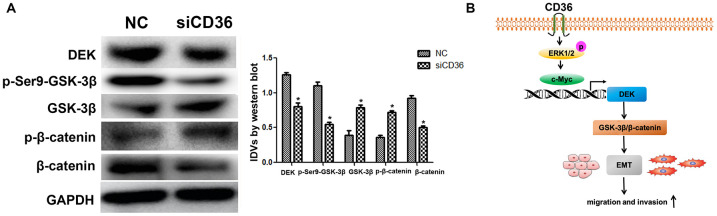
**CD36 upregulates GSK-3β/β-catenin signaling by enhancing DEK expression in GC cells.** (**A**) Western blot analysis of GSK-3β/p-Ser9-GSK-3β and β-catenin/p-β-catenin in SNU-216 cells transfected with NC/siCD36. Data are presented as the mean ± SD.*p < 0.05 vs. NC group. GAPDH was used as endogenous control. (**B**) Schematic representation of the proposed mechanism by which CD36 overexpression induces EMT in GC cells.

The Wnt/β-catenin pathway is a classical regulator of EMT. Previous studies have reported that silencing DEK inhibits the Wnt/β-catenin signaling pathway by mediating GSK-3β phosphorylation in cervical cancer [21]. To assess whether CD36 induces EMT via Wnt/β-catenin signaling in GC cells, we examined the effect of CD36 depletion on Wnt/β-catenin pathway markers. As shown in Figure 6A, compared with control cells, SNU-216 cells transfected with siCD36 exhibited a dramatic increase in both GSK-3β and p-β-catenin protein expression concurrently with a significant decrease in both p-Ser9-GSK-3β and β-catenin levels. Consistent with the results of DEK knockdown and overexpression experiments described above, these results imply that CD36 promotes DEK expression to stimulate GSK-3β/β-catenin signaling in GC cells.

## DISCUSSION

Although several studies have shown that CD36 expression is associated with tumorigenesis, its precise role in this process remains to be fully determined. Early studies suggested that the expression of CD36 was negatively associated with progression and metastasis of breast cancer and was decreased by estradiol in hormone-dependent breast cancer cell lines [22, 23]. In recent years, however, CD36 has been found to be involved in the progression of various tumors. For instance, CD36 was reported in 2017 as the first molecular marker specific for tumor invasion and metastasis in oral squamous cell carcinoma [12, 24, 25]. CD36 expression has been proposed to predict poor survival in GC [17], an association that we validated on TCGA and GEO data and in our internal cohort of 79 Chinese patients. However, to date only one study has described the molecular mechanisms underlying the tumor-promoting effects of CD36 in GC. In the referred study, Jiang et al. reported that fatty acid-induced hyper-O-GlcNAcylation promotes CD36 expression to drive GC metastasis [26]. In this study, we demonstrated the essential role of CD36 in the migration and invasion of GC cells. Moreover, by combining mRNA array data from CD36 knockdown GC cells and MSigDB Hallmark-GSEA analysis, we identified DEK as a key downstream gene linking CD36 overexpression with EMT onset.

DEK is a multifunctional nuclear protein involved in the regulation of chromatin remodeling and gene transcription [27]. Reflecting its oncogenic potential, it is highly expressed in various malignant tumors (*e.g.* colon cancer, hepatocellular cancer, and non-small cell lung cancer) in association with malignant progression, EMT, and metastasis [21, 28–30]. However, the potential role of DEK in the migration and invasion of GC cells has not been determined. In this study, DEK knockdown inhibited migration and invasion in cultured GC cells. Moreover, based on the presence of DEK in the Myc-Targets-V1 gene set revealed by Hallmark-GSEA analysis, we showed that c-Myc can bind to the DEK promoter and accelerate its expression in GC cells. To our knowledge, this is the first study to demonstrate that c-Myc exerts transcriptional regulation of DEK expression in GC cells.

The Wnt/β-catenin pathway is a classic regulator of EMT. Our study showed, for the first time, that CD36 levels positively regulate EMT in GC cells by activating the DEK/GSK-3β/β-catenin axis. This finding is thus consistent with that of a previous study in cervical cancer cells, which demonstrated that DEK silencing inhibited tumorigenesis and metastasis by repressing p-Ser9-GSK-3β, leading to β-catenin degradation [21]. Although EMT induction appears to be a hallmark of the metastasis-promoting actions of CD36, the specific mechanism(s) remain controversial. For example, analysis of CD36^+^ cells showed that EMT-associated genes were expressed at lower levels in primary lesions and lymph node metastases in cases of oral squamous cell carcinoma [12]. These data suggested that, at least in this tumor type, the mechanism by which CD36 promotes metastasis may not be related to EMT. In contrast, studies on hepatocellular cancer (HCC) cells indicated that EMT was closely associated with CD36 expression via Wnt and TGF-β signaling pathways [15]. Another study, conducted also in HCC cells, reported that upregulation of EMT markers resulted from activation of the MEK/ERK and PI3K/AKT pathways through cartilage oligomeric matrix protein (COMP)/CD36 signaling [11]. Meanwhile, in cervical cancer cells CD36 has been shown to promote EMT by interacting with TGF-β [16]. Altogether, the evidence available suggests that tumor heterogeneity may account for the diversity of mechanisms by which CD36 promotes metastasis in different tumor types.

In summary, our study demonstrated that CD36 promotes GC cell migration and invasion by inducing c-Myc-dependent DEK transcription, GSK-3β/β-catenin pathway activation, and EMT (Figure 6B). These data provide a better understanding of the signal transduction pathways conferring metastatic potential to GC cells and suggest that CD36 may serve as a novel target in GC.

## MATERIALS AND METHODS

### Bioinformatics analyses

Analysis of survival outcome of CD36 expression in GC patients was performed using The Cancer Genome Atlas (TCGA) (https://tcga-data.nci.nih.gov) and the Gene Expression Omnibus (GEO) (http://www.ncbi.nlm.nih.gov/geo/) databases. Hallmark gene sets were downloaded from the Molecular Signatures Database (MSigDB) from the Gene Set Enrichment Analysis (GSEA) website (http://www.broadinstitute.org/gsea/msigdb/). An mRNA expression microarray constructed from SNU-216 cells transfected with a CD36-targeted siRNA or a negative control siRNA was prepared and analyzed by Oebiotech Co. (Shanghai, China).

### Cell culture and clinical GC samples

All cells were purchased from the Shanghai Chinese Academy of Sciences and cultured at 37°C and 5% CO_2_ in RPMI-1640 medium (Gibco, MA, USA) supplemented with 10% fetal bovine serum (Thermo Scientific, MA, USA) and 100 units/ml penicillin-streptomycin. GC FFPE specimens (resected at least 20 mm away from the margin with normal tissue) were obtained from the Pathology Department of the First Hospital of China Medical University. All tumor samples were histopathologically confirmed as GC.

### Immunohistochemistry

Immunohistochemical (IHC) staining was performed as described by us previously [31]. An anti-CD36 antibody (MAB19554, R&D, Minneapolis, USA) was applied to FFPE GC specimens. Based on IHC staining intensity scores assigned independently by two pathologists, samples were classified as CD36-negative or CD36-positive.

### Analysis of CD36 expression by flow cytometry

Membrane expression of CD36 was detected by flow cytometry (BD Accuri C6 instrument; BD Biosciences, San Jose, CA, USA) following incubation with a PE-conjugated anti-human CD36 antibody (555455, BD) for 20 min at 4°C. An isotype control antibody was used as negative staining control.

### Quantitative real-time PCR

Total RNA was extracted from cultured cells using Trizol reagent (Invitrogen, Carlsbad, CA, USA). RNA quantity and purity were determined by absorbance measurements at 260 nm and 260/280 nm, respectively, with a NanoDrop ND-100 spectrophotometer (NanoDrop Technologies, Rockland, DE, USA). A PrimeScript^TM^ RT Reagent Kit (Takara, Japan) was used for mRNA reverse transcription. Relative mRNA expression was calculated via the 2^−ΔΔCT^ method after normalization to 18S rRNA. qRT- PCR was carried out on an ABI PRISM 7500 system using SYBR^®^ Premix Ex Taq^TM^ II (Takara, Japan). The following PCR primers were used:

CD36 forward: 5′- TGCAAGTCCTGATGTTTCAGA -3′;

reverse: 5′- TGGCTTGACCAATAGGTTGAC -3′.

DEK forward: 5′-AGGCACTGTGTCCTCATTAA-3′;

reverse: 5′- TCTGACAGAATTTCAGGACA-3′.

c-Myc forward: 5′- TCAAGAGGCGAACACACAAC-3′;

reverse: 5′- GGCCTT- TTCATTGTTTTCCA-3′.

18S forward: 5′-CCCGGGGAGGTAGTGACGAAAAAT-3′;

reverse: 5′-CGCCCGCCCGCTCCCAAGAT-3′.

### Transient transfection

The siRNAs against CD36, c-Myc, and DEK and their corresponding negative controls (NC) were designed and synthesized by RiboBio (Guangzhou, China). Since siCD36-1, siDEK-2, and sic-Myc-2 showed better transfection efficiency than siCD36-2, siDEK-1, and sic-Myc-1, respectively (Figure 2 and Supplementary Figure 2), siRNA-mediated silencing was performed with siCD36-1, siDEK-2, and sic-Myc-2. Transfection was established using Lipofectamine 2000 (Invitrogen, CA, USA) according to the manufacturer’s protocol. Overexpression plasmids containing the complete coding sequence for CD36 (pcDNA3.1-CD36) or DEK (pcDNA3.1-DEK), as well as empty pcDNA 3.1 vectors (NC) were purchased from GeneChem (Shanghai, China) (Figure 2 and Supplementary Figure 2). The corresponding sequences were:

siCD36-1:5’- CACUAUCAGUUGGAACAGATT-3’;

siCD36-2:5’- GGACCAUUGGUGAUGAGAATT-3’;

siDEK-1:5’- CCAUUGCCGAAAUCUAAAATT-3’;

siDEK-2:5’-GAUCAGGUGUAAAUAGUGATT-3’;

sic-Myc-1:5’-GGCGAACACACAACGUCUUTT-3’;

sic-Myc-2:5’-CCACACAUCAGCACAACUATT-3’;

NC siRNA: 5′-AATTCTCCGAACGTGTCACGT-3′.

### Migration and invasion assays

Cell migration and invasion assays were performed using Transwell chambers (8-μm pore size membrane; Corning, USA). Non-coated membranes were used for migration experiments, whereas Matrigel-coated membranes were used to assay cell invasion. Briefly, 2 × 10^4^ cells were plated in serum-free medium onto the upper compartment of the chamber. As chemo-attractant, medium containing 5% fetal bovine serum was added to the lower compartment. The plates were incubated for 24 h at 37°C, after which the cells that migrated to the lower surface of the membranes were fixed, stained with trypan blue, and counted in 5 random fields under a microscope (200x magnification). Each experiment was repeated three times.

### Western Blot Analysis

Western blot analysis was conducted as previously described [32], in samples collected 48 hours after transfection. The following primary antibodies were used: CD36 (R&D, Minneapolis, USA), DEK (Abcam), c-myc (Santa Cruz Biotechnology, Inc., Dallas, TX, USA), phospho-ERK, ERK, E-cadherin, Vimentin, Snail, ZEB1, GSK-3β, p-Ser9-GSK-3β, β-catenin, phospho-β-catenin, and GAPDH (Cell Signaling Technology). Secondary goat anti-rabbit and goat anti-mouse antibodies were purchased from Santa Cruz Biotechnology. The corresponding catalog numbers and dilutions are shown in Supplementary Table 1.

### Immunofluorescence

Immunofluorescence was performed based on standard procedures [14]. The cells were transfected with siCD36 or NC for 48 hours, fixed in 3.3% paraformaldehyde for 15 min, permeabilized with 0.2% Triton X-100 for 5 min, blocked with 5% bovine serum albumin (BSA) for 1 h, and incubated with anti-E-cadherin and anti-vimentin antibodies overnight at 4°C. Secondary Alexa Fluor 594- and Alexa Fluor 488-conjugated antibodies were then applied for 1 h at room temperature in the dark. DAPI (4′6′-diamidino-2 phenylindole) was used to stain nuclei. After mounting (Slow Fade Antifade Kit; Molecular Probes, Eugene, OR, USA), the cells were visualized by fluorescence microscopy (BX61, Olympus, Japan).

### Luciferase reporter assay

Luciferase reporter gene assays were performed using the Dual-Luciferase Reporter Assay System (Promega, USA), according to the manufacturer’s instructions. Plasmids containing the predicted wild-type (WT) or a mutant (Mut) c-Myc binding site on the DEK gene were obtained from OBiO Technology (Shanghai, China). The Mut-DEK promoter luciferase reporter plasmid was generated by deleting the c-Myc binding sequence (bp 678-689), as shown in Figure 5B, 5D.

### Statistics

Statistical analyses were carried out using SPSS 17.0 software (SPSS, Chicago, IL, USA) or R software (version 3.2.3). Continuous data are expressed as the mean ± standard deviation (SD) obtained from three independent experiments. Comparisons between treatments were made using Student’s t-test. The Kaplan–Meier method and the log-rank test were used to compare survival curves. P < 0.05 was considered significant. All experiments were performed at least in triplicate.

## Supplementary Material

Supplementary Figures

Supplementary Table 1
