# Correction for: CD36 upregulates DEK transcription and promotes cell migration and invasion via GSK-3β/β-catenin-mediated epithelial-to-mesenchymal transition in gastric cancer

**DOI:** 10.18632/aging.204046

**Published:** 2022-04-29

**Authors:** Jin Wang, Ti Wen, Zhi Li, Xiaofang Che, Libao Gong, Zihan Jiao, Xiujuan Qu, Yunpeng Liu

**Affiliations:** 1 Department of Medical Oncology, The First Hospital of China Medical University, Shenyang, 110001, China; 2 Key Laboratory of Anticancer Drugs and Biotherapy of Liaoning Province, The First Hospital of China Medical University, Shenyang, 110001, China; 3 Liaoning Province Clinical Research Center for Cancer, The First Hospital of China Medical University, Shenyang, 110001, China; 4 Key Laboratory of Precision Diagnosis and Treatment of Gastrointestinal Tumors, Ministry of Education, The First Hospital of China Medical University, Shenyang, 110001, China

**Keywords:** CD36, epithelial-to-mesenchymal transition, gastric cancer, DEK

Original article: Aging. 2021; 13:1883–1897.  . https://doi.org/10.18632/aging.103985

**This article has been corrected: Figure 4** was replaced because of errors in the images used in panel 4E. The SNU-216 NC/migration and invasion images in panel 2E were mistakenly reused as the SNU-216 invasion/NC and SNU-216 migration/siCD36 images, respectively, in panel 4E. In addition, the SNU-216 invasion/siDEK image in panel 4D was mistakenly reused as the SNU-216 invasion/si-CD36+pcDNA3.1-DEK image in panel 4E. The new images for SNU-216 invasion/NC, SNU-216 invasion/si-CD36+pcDNA3.1-DEK and SNU-216 migration/siCD36 in the new panel 4E are from the original set of experiments. These corrections do not change the content of the publication and do not affect the conclusions drawn from this research.

The correct **Figure 4** is presented below.

**Figure 4:**
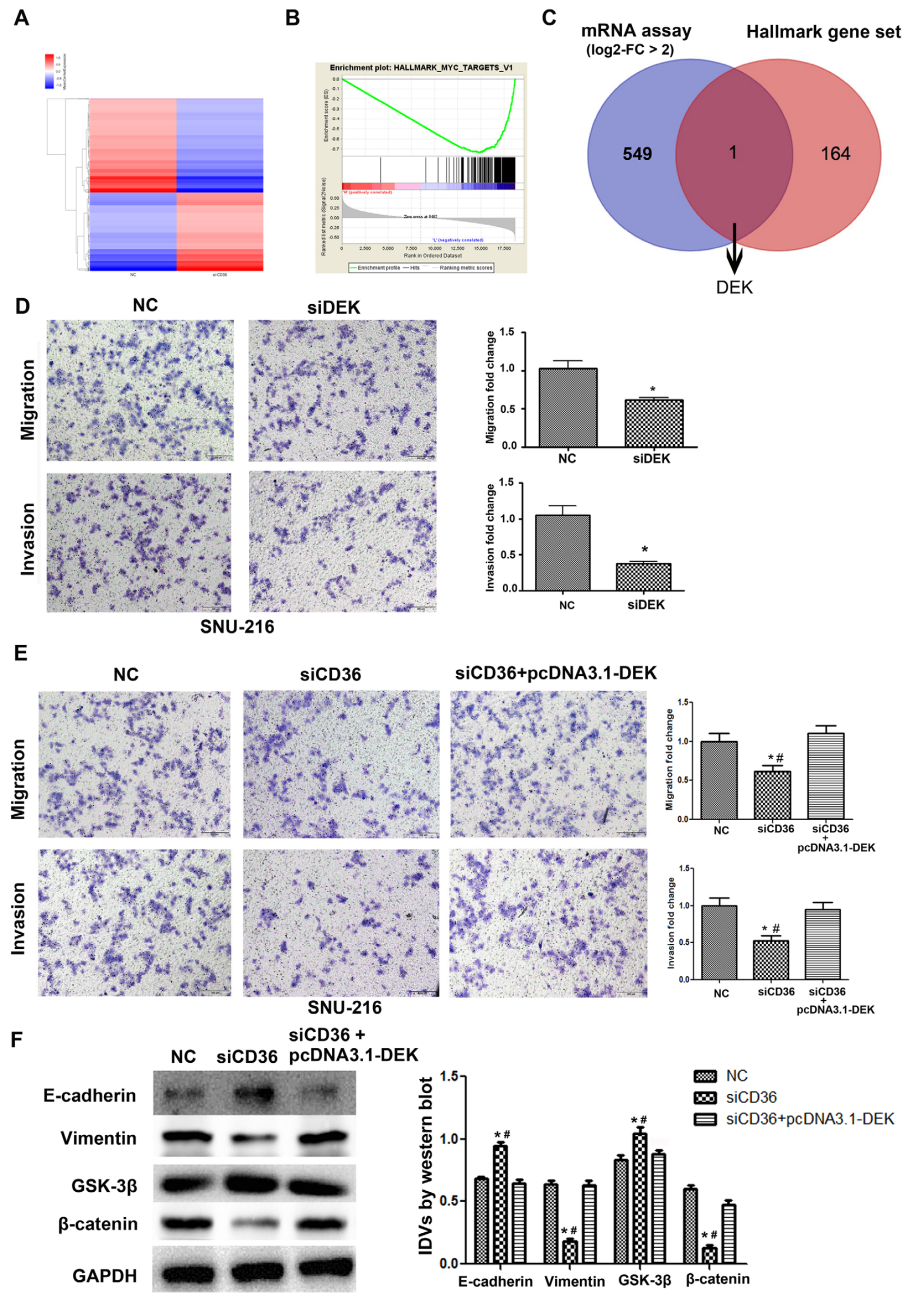
**CD36 positively regulates DEK to promote migration and invasion in GC cells.** (**A**) Heatmap of log2-fold gene expression changes in SNU-216 cells transfected with NC/siCD36. (**B**) Enrichment of genes within the Myc-targets-V1 pathway in the CD36-down-regulated Hallmark gene set. (**C**) Venn diagram of candidate genes downstream of CD36 obtained from combined mRNA array and MSigDB Hallmark-GSEA analyses. (**D**) Results of Transwell migration and invasion assays conducted on SNU-216 cells transfected with NC/siDEK. (**E**) Results of Transwell migration and invasion assays carried out in SNU-216 cells transfected with NC/siCD36/siCD36 + pcDNA3.1-DEK. (**F**) Western blot analysis of EMT and GSK-3β/β-catenin signaling markers in SNU-216 cells transfected with NC/siCD36/siCD36 + pcDNA3.1-DEK. Data are presented as the mean ± SD. For D-F, *p < 0.05 vs. NC group; #*p* < 0.05 vs. siCD36 + pcDNA3.1-DEK group. GAPDH was used as endogenous control.

