# Supplementary material for: CD36 upregulates DEK transcription and promotes cell migration and invasion via GSK-3β/β-catenin-mediated epithelial-to-mesenchymal transition in gastric cancer
Source: Aging (Albany NY). 2020 Nov 21;13(2):1883–97. doi: 10.18632/aging.103985 (PMC7880392; doi:10.18632/aging.103985)
Supplement: Supplementary Figures [file aging-13-103985-s001.pdf]

SUPPLEMENTARY FIGURES

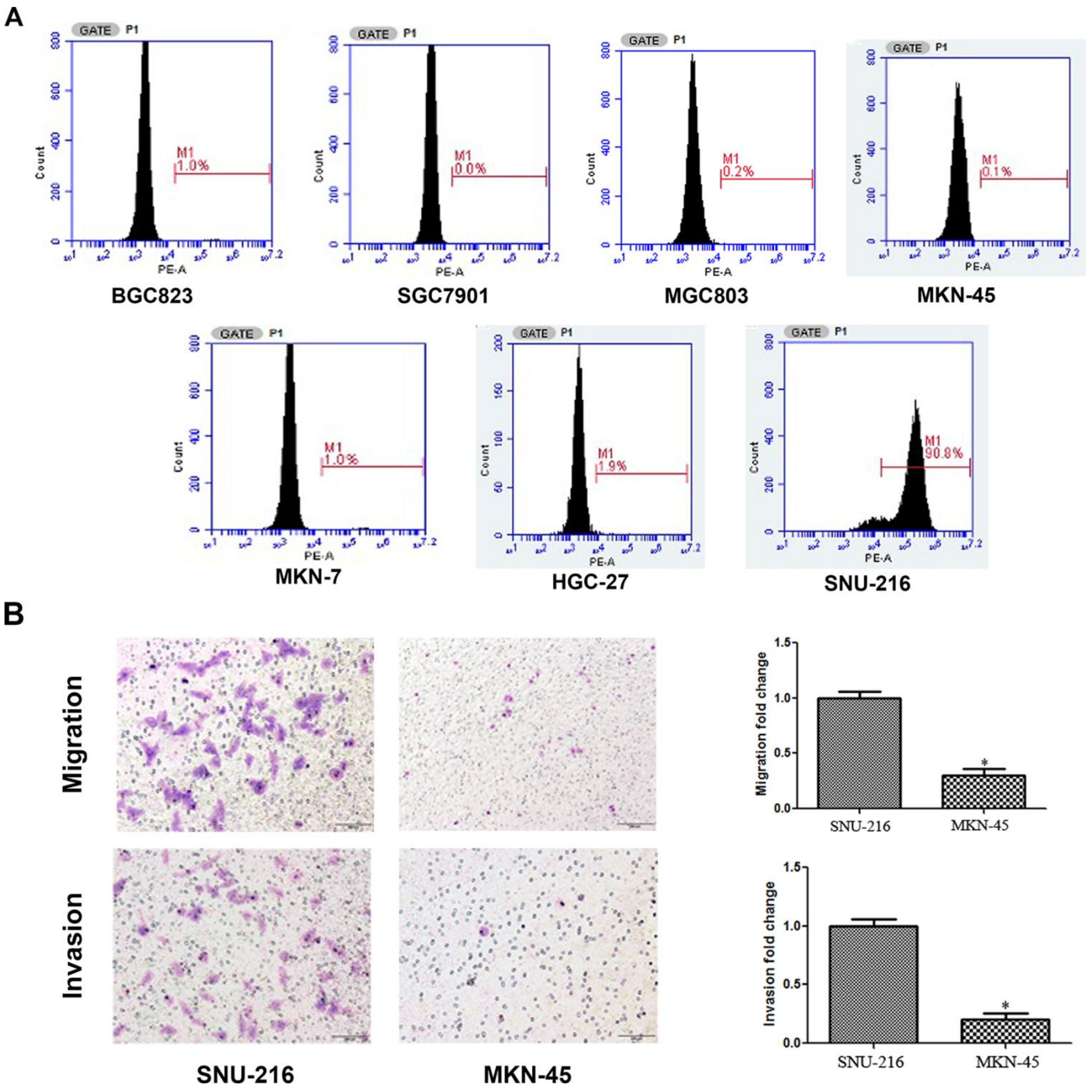

Supplementary Figure 1. (**A**) Flow cytometry analysis of cell membrane CD36 expression in seven GC cell lines. (**B**) Results of Transwell migration and invasion assays conducted on SNU-216 and MKN-45 cells. Data are presented as the mean ± SD.*$p < 0.05$ vs. NC group.

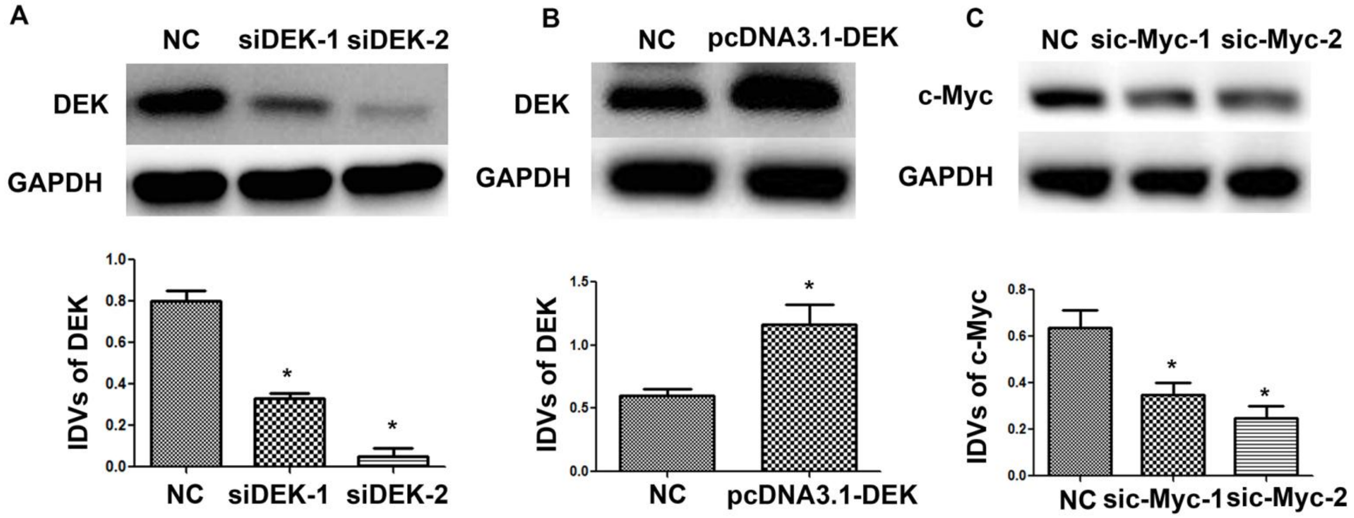

Supplementary Figure 2. (**A**) Western blot analysis of the transfection efficiencies of NC/pcDNA3.1-DEK plasmids. (**B**) Western blot analysis of the transfection efficiencies of NC/siDEK. (**C**) Western blot analysis of the transfection efficiencies of NC/sic-Myc. Data are presented as the mean ± SD. *p < 0.05 vs. NC group. GAPDH was used as endogenous control. IDV: integrated densitometric value.
