# Supplementary material for: CD36 upregulates DEK transcription and promotes cell migration and invasion via GSK-3β/β-catenin-mediated epithelial-to-mesenchymal transition in gastric cancer
Source: Aging (Albany NY). 2020 Nov 21;13(2):1883–97. doi: 10.18632/aging.103985 (PMC7880392; doi:10.18632/aging.103985)
Supplement: Supplementary Table 1 [file aging-13-103985-s002.pdf]

SUPPLEMENTARY TABLE

Supplementary Table 1. Antibody information.

Application	Protein symbol	Antibody source	Catalog number
Flow cytometry	CD36	BD	555455
Western blot, Immunohistochemistry	CD36	R&D system	MAB19554
Western blot	DEK	Abcam	ab166624
Western blot	phospho-ERK	Cell Signaling Technology	4370S
Western blot	ERK	Cell Signaling Technology	9102S
Western blot	c-myc	Santa Cruz	sc-40
Western blot, Immunofluorescence	E-cadherin	Cell Signaling Technology	3195S
Western blot, Immunofluorescence	Vimentin	Cell Signaling Technology	5741S
Western blot	Snail	Cell Signaling Technology	3879S
Western blot	ZEB1	Cell Signaling Technology	3396S
Western blot	GSK3β	Cell Signaling Technology	9315S
Western blot	p-Ser9-GSK3β	Cell Signaling Technology	9323S
Western blot	β-catenin	Cell Signaling Technology	9581S
Western blot	p-β-catenin	Cell Signaling Technology	9561S
Western blot	GAPDH	Cell Signaling Technology	2118S
